# Supplementary material for: Identification of Mycoplasma Species in Cattle Associated with Bovine Respiratory Disease Mortality
Source: Microorganisms. 2024 Nov 16;12(11):2340. doi: 10.3390/microorganisms12112340 (PMC11596787; doi:10.3390/microorganisms12112340)
Supplement: Supplementary file 1 [file microorganisms-12-02340-s001.zip › Table S1 Demographic data, including sex, age, and sampling location, recorded for each animal that tested positive for Mycoplasma spp.pdf]

Table S1 Demographic data, including sex, age, and sampling location, recorded for each animal that tested positive for *Mycoplasma* spp.

animal ID	sampling date	sex	age (months)	weigh (kg)	municipality	province
48902/20	June 2020	F	12	350	Vigone	Torino
76314/20	October 2020	F	1	45	Pralormo	Torino
62214/20	September 2020	M	10	270	Cavour	Torino
50470/20	July 2020	M	4	100	San Sebastiano Po	Torino
5245/20	January 2020	F	24	800	Rivarolo	Torino
72746/20	October 2020	M	3	40	Favria	Torino
44261/21	May 2021	M	6	150	Rivoli	Torino
686/21	January 2021	M	6	150	Cavour	Torino
96990/21	November 2021	M	12	400	Rivoli	Torino
33779/21	April 2021	F	3	80	Cigliano	Vercelli
74863/21	September 2021	F	2	50	Grugliasco	Torino
97257/21	November 2021	M	12	330	Druento	Torino
70066/21	September 2021	M	6	200	Leini	Torino
107261/21	December 2021	M	4	130	Scalenghe	Torino
31019/21	April 2021	M	12	350	Chivasso	Torino
95124/21	November 2021	M	12	300	Cavour	Torino
73845/21	September 2021	M	5	150	Garbagna	Alessandria
5258/21	January 2021	M	2	40	Vische	Torino
27117/21	March 2021	M	1	35	Grugliasco	Torino
86346/21	October 2021	F	24	600	None	Torino
11599.1/22	February 2022	F	36	700	Cavour	Torino
11599.2/22	February 2022	M	24	580	Cavour	Torino
10395/22	February 2022	F	24	600	Cavour	Torino
18123/22	February 2022	F	12	300	Grugliasco	Torino
27549/22	March 2022	M	1	35	Canale	Cuneo
92270/22	November 2022	M	10	250	Riva presso Chieri	Torino
5674.1/22	January 2022	M	8	300	Cavour	Torino
5674.2/22	January 2022	M	8	300	Cavour	Torino
62678.1/22	July 2022	F	7	250	Savigliano	Cuneo
62678.2/22	July 2022	F	7	250	Savigliano	Cuneo
18119/22	February 2022	M	3	50	Grugliasco	Torino
18952/22	February 2022	F	3	90	Revello	Cuneo
85837/22	October 2022	M	5	130	Macello	Torino
44807/22	May 2022	F	12	280	Barge	Cuneo
107173/22	December 2022	M	3	120	Villafranca Piemonte	Torino
97248/22	November 2022	F	4	100	San secondo di Pinerolo	Torino
31031/22	April 2022	F	5	200	Lessolo	Torino
87327/22	October 2022	F	5	200	Grugliasco	Torino
99323/22	December 2022	M	4	150	Revello	Cuneo
101463/22	December 2022	M	5	200	Brà	Cuneo
7880/22	January 2022	F	108	500	Campertogno	Vercelli
21353/23	March 2023	M	4	130	Buriasco	Torino
24540/23	March 2023	F	3	100	Scarnafigi	Cuneo
28308/23	March 2023	M	4	150	Fossano	Cuneo
28315/23	March 2023	F	3	100	Carmagnola	Torino
56395/23	June 2023	F	12	400	Ruffia	Cuneo

60338/23	June 2023	F	3	100	Villanova d'Asti	Asti
21637/23	March 2023	M	18	600	None	Torino
39903/23	April 2023	F	4	110	Cunico	Asti
39914/23	April 2023	M	1	45	Cunico	Asti
39920/23	April 2023	F	0	35	Chivasso	Torino
45084/23	May 2023	F	12	330	Alessandria	Alessandria
51334/23	May 2023	M	4	100	Agrate Conturbia	Novara
52521/23	May 2023	M	12	650	Carema	Torino
