# Supplementary material for: Identification of Mycoplasma Species in Cattle Associated with Bovine Respiratory Disease Mortality
Source: Microorganisms. 2024 Nov 16;12(11):2340. doi: 10.3390/microorganisms12112340 (PMC11596787; doi:10.3390/microorganisms12112340)
Supplement: Supplementary file 1 [file microorganisms-12-02340-s001.zip › Table S2 Strains used for the phylogenetic tree construction.pdf]

Table S3. Strains used for the phylogenetic tree construction.

Strain/Clone ID	GenBank Accession Number	Species	Origin
5245.2020	PQ498316	*Ureaplasma diversum*	This study
48902.2020	PQ498315	*Mycoplasma bovis*	This study
50470.2020	PQ498314	*Mycoplasma dispar*	This study
62214.2020	PQ498313	*Mycoplasma alkalescens*	This study
72746.2020	PQ498312	*Mycoplasma dispar*	This study
76314.2020	PQ498311	*Ureaplasma diversum*	This study
686.2021	PQ498310	*Ureaplasma diversum*	This study
5258.2021	PQ498309	*Mycoplasma dispar*	This study
27117.2021	PQ498308	*Mycoplasma bovis*	This study
31019.2021	PQ498307	*Mycoplasma bovis*	This study
33779.2021	PQ498306	*Ureaplasma diversum*	This study
44261.2021	PQ498305	*Mycoplasma bovis*	This study
70066.2021	PQ498304	*Ureaplasma diversum*	This study
73845.2021	PQ498303	*Mycoplasma dispar*	This study
74863.2021	PQ498302	*Mycoplasma bovis*	This study
86346.2021	PQ498301	*Mycoplasma bovis*	This study
95124.2021	PQ498300	*Mycoplasma bovis*	This study
96990.2021	PQ498299	*Mycoplasma bovis*	This study
97257.2021	PQ498298	*Mycoplasma bovis*	This study
107261.2021	PQ498297	*Mycoplasma bovis*	This study
5674.1.2022	PQ498296	*Mycoplasma bovis*	This study
5674.2.2022	PQ498295	*Mycoplasma bovis*	This study
7880.2022	PQ498294	*Mycoplasma bovigenitalium*	This study
10359.2022	PQ498293	*Mycoplasma alkalescens*	This study
11599.1.2022	PQ498292	*Mycoplasma canadense*	This study
11599.2.2022	PQ498291	*Mycoplasma canadense*	This study
18119.2022	PQ498290	*Mycoplasma bovis*	This study
18123.2022	PQ498289	*Mycoplasma dispar*	This study
18952.2022	PQ498288	*Mycoplasma dispar*	This study
27549.2022	PQ498287	*Ureaplasma diversum*	This study
31031.2022	PQ498286	*Mycoplasma arginini*	This study
44807.2022	PQ498285	*Mycoplasma hyopharyngis*	This study
62678.1.2022	PQ498284	*Mycoplasma bovirhinis*	This study
62678.2.2022	PQ498283	*Mycoplasma bovirhinis*	This study
85837.2022	PQ498282	*Mycoplasma dispar*	This study
87327.2022	PQ498281	*Mycoplasma dispar*	This study
92270.2022	PQ498280	*Mycoplasma bovis*	This study
97248.2022	PQ498279	*Mycoplasma dispar*	This study
99323.2022	PQ498278	*Mycoplasma bovis*	This study
101463.2022	PQ498277	*Mycoplasma dispar*	This study
107173.2022	PQ498276	*Mycoplasma dispar*	This study
21353.2023	PQ498275	*Mycoplasma alkalescens*	This study
21637.2023	PQ498274	*Mycoplasma alkalescens*	This study
24540.2023	PQ498273	*Mycoplasma bovis*	This study
28308.2023	PQ498272	*Mycoplasma bovis*	This study
28315.2023	PQ498271	*Mycoplasma bovis*	This study
39903.2023	PQ498270	*Mycoplasma arginini*	This study
39914.2023	PQ498269	*Mycoplasma bovis*	This study
39920.2023	PQ498268	*Mycoplasma bovis*	This study
45084.2023	PQ498267	*Mycoplasma bovis*	This study
51334.2023	PQ498266	*Mycoplasma dispar*	This study
52521.2023	PQ498265	*Mycoplasma dispar*	This study
56395.2023	PQ498264	*Mycoplasma bovirhinis*	This study

60338.2023	PQ498263	*Mycoplasma bovis*	This study
A417	NR025878	*Ureaplasma diversum*	GenBank (field strain)
ATCC 27618	NR041710	*Ureaplasma urealyticum*	GenBank (reference strain)
G37	NR026155	*Mycoplasma genitalium*	GenBank (field strain)
ATCC 29342	AF132741	*Mycoplasma pneumoniae*	GenBank (reference strain)
PG51	LC158831	*Mycoplasma alkalescens*	GenBank (field strain)
275C	LC158835	*Mycoplasma canadense*	GenBank (field strain)
G230	NR041743	*Mycoplasma arginini*	GenBank (field strain)
ATCC 23114	JN935871	*Mycoplasma hominis*	GenBank (reference strain)
462/2 (T)	AF412979	*Mycoplasma dispar*	GenBank (field strain)
-	E02783	*Mycoplasma hyopneumoniae*	GenBank (field strain)
M165/69	U44768	*Mycoplasma bovoculi*	GenBank (field strain)
NBRC 14857	AB680687	*Mycoplasma bovirhinis*	GenBank (reference strain)
NBRC 14846	AB680678	*Mycoplasma canis*	GenBank (reference strain)
-	U58997	*Mycoplasma hyopharyngis*	GenBank (field strain)
PG11	NR179203	*Mycoplasma bovigenitalium*	GenBank (field strain)
PG45	NR102850	*Mycoplasma bovis*	GenBank (field strain)
